# Analysis of the Functional Status Score for the Intensive Care Unit and its correlation with measures of muscle strength in critically ill patients during hospitalization in the intensive care unit

**DOI:** 10.62675/2965-2774.20250197

**Published:** 2025-01-30

**Authors:** Gabriela de Sousa Martins, Katryne Holanda Silva, William Rafael Almeida Moraes, Eduardo Yoshio Nakano, Joanlise Marco de Leon Andrade, Laura Maria Tomazi Neves, Graziella França Bernardelli Cipriano

**Affiliations:** 1 Universidade de Brasília Postgraduate Program in Health Sciences and Technologies Brasília DF Brazil Postgraduate Program in Health Sciences and Technologies, Universidade de Brasília - Brasília (DF), Brazil.; 2 Universidade de Brasília Postgraduate Program in Rehabilitation Sciences Brasília DF Brazil Postgraduate Program in Rehabilitation Sciences, Universidade de Brasília - Brasília (DF), Brazil.; 3 Universidade Federal do Pará Postgraduate Program in Movement Science Belém PA Brazil Postgraduate Program in Movement Science, Universidade Federal do Pará - Belém (PA), Brazil.; 4 Universidade de Brasília Department of Statistics Brasília DF Brazil Department of Statistics, Universidade de Brasília - Brasília (DF), Brazil.

Advancements in intensive care have led to a reduction in mortality rates in intensive care units (ICUs), resulting in increased survival of critically ill patients but also increased challenges in health care and in the long-term recovery of survivors.^(1,2)^ Early measurement of functional status (FS) and muscle strength (MS) and follow-up measurements in the ICU are essential for identifying patients with physical decline, monitoring the effectiveness of rehabilitation interventions and observing the evolution of recovery.^(2)^ A scoping review^(3)^ reported the existence of approximately 60 instruments for assessing FS, although no gold standard has been established.

In this context, we evaluated the progression of FS and MS during the duration of ICU stays. We determined the association of the Functional Status Score for the Intensive Care Unit (FSS-ICU) with MS. We assessed the predictive value of MS measurements for patient independence in the FSS-ICU at awakening. We performed a prospective observational cohort study in which patients were followed from awakening until discharge from the ICU. Functional status was assessed with the FSS-ICU, and MS was assessed with the Medical Research Council-Sum Score (MRC-SS) and handgrip strength (HGS). The present study was approved by the Committee for Ethics in Human Research of the *Fundação de Ensino e Pesquisa em Ciências da Saúde*/ESCS (CAEE 30442514.7.0000.5553).

The assessments were performed upon awakening and upon discharge from the ICU, and the results were compared via the paired Wilcoxon test. Associations were determined via Spearman's correlation, and a receiver operating characteristic (ROC) curve was used to determine the cutoff points for MS. A complete description of the methods can be found in the Supplementary Material (Table S1 and Figure S1). The sample consisted of 48 participants who were predominantly males (62%), with a mean age of 49 ± 16 years and a median (interquartile deviation [IQD]) duration of ICU stay of 10 (14) days (Table 1). This results are similar to those identified in a review study of 113 Brazilian intensive care units.^(4)^

**Table 1:** Characteristics of critically ill patients in the study cohort

Characteristics			
Baseline characteristics			
	Males		30 (62)
	Age (years)		49 ± 16
	BMI (kg/m^2^)		25 ± 5
Risk factors			
	Arterial hypertension		27 (57)
	Diabetes mellitus		13 (27)
	Alcoholism		24 (50)
	Smoking		30 (62)
Hospitalization characteristics			
	APACHE		18 (13)
Cause of ICU admission			
	Respiratory		10 (21)
	Cardiovascular		9 (19)
	Infectious		9 (19)
	Postoperative		8 (17)
	Neurological		4 (8)
	Others		7 (16)
Organic dysfunctions			
	Sepsis		16 (33)
	Hemodialysis		13 (27)
	Hematological replacement		9 (19)
	Acquired muscle weakness		32 (68)
MV usage			36 (75)
	MV days		6 (10)
Medications during ICU stay			
	Corticosteroids		33 (69)
		Days of use	4 (9)
	Vasoactive drugs		30 (62)
		Days of use	3 (8)
	Diuretics		36 (75)
		Days of use	7 (12)
	Antibiotics		45 (94)
		Days of use	9 (7)
	Sedatives		35 (73)
		Days of use, median	3 (7)
	Muscle blockers		10 (21)
		Days of use	0
Evaluation of functional status			
	FSS-ICU total score at awakening		15 (17)
	FSS-ICU maximum total score		26 (17)*
Assessment of muscle strength			
	MRC-SS total score at awakening		44 (15)
	MRC-SS maximum total score		52 (12)†
		HGS of dominant hand, total score at awakening	16 (12)
		HGS of dominant hand, maximum total score	20 (13)‡
Days of hospitalization before ICU			2 (6)
Days of ICU stay			10 (14)
Days between ICU admission and awakening			7 (8)

BMI - body mass index; APACHE II - Acute Physiology and Chronic Health Evaluation II; MV - mechanical ventilation; ICU - intensive care unit; FSS-ICU - Functional Status Score for the Intensive Care Unit; MRC-SS - Medical Research Council - Sum Score; HGS - handgrip strength.

* value of the FSS-ICU at admission *versus* at discharge = < 0.001

† p value of the MRC-SS at admission *versus* at discharge test = < 0.001

‡ p value of HGS of the dominant hand at admission *versus* at discharge = 0.002. Data are expressed as n (%), mean ± standard deviation and median (interquartile deviation).

Comparisons were performed using the paired Wilcoxon test with a significance level of p ≤ 0.05.

During the ICU stay, we observed differences in the total FSS-ICU score between awakening and discharge and between the pre-ambulation and ambulation scores (p < 0.001), similar to the differences observed in MS as evaluated by the MRC-SS (p = 0.002) and HGS (p = 0.001). Most patients were right-handed (71%) (Table 1). Similar results were observed in other cohort studies conducted in Australia,^(5)^ the United States^(6)^ and Brazil.^(7)^

The FSS-ICU score was significantly correlated with the MRC-SS (rho = 0.74 and rho = 0.75) and HGS (rho = 0.57 and rho = 0.42) at awakening and at discharge (Figure 1A and 1B). The MRC-SS cutoffs for independence in the FSS-ICU were 49 points (area under the ROC curve [AUC] = 0.912; 95%CI = 0.826 − 0.998; p < 0.001) for pre-ambulation and 57 points (AUC = 0.923; 95%CI = 0.838 − 1.000; p = 0.001) for ambulation. For HGS, the cutoff values were 16kg/f (AUC = 0.769; 95%CI = 0.610 − 0.929; p = 0.007) and 18kg/f (AUC = 0.720; 95%CI = 0.511 − 0.929; p = 0.008) (Figure 1C and 1D).

**Figure 1:**
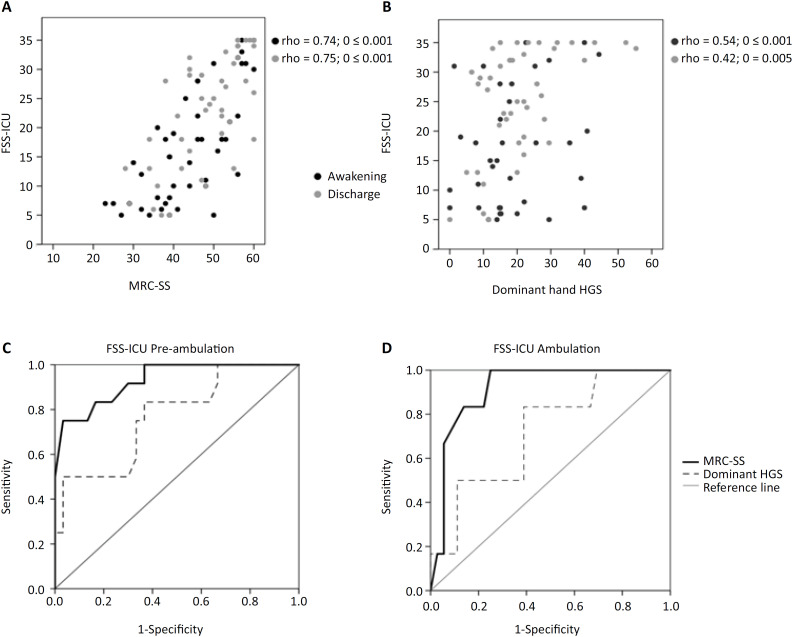
Associations between functional status and muscle strength measurements and the predictive value of muscle strength for performance in the Functional Status Score for the Intensive Care Unit.

(A) and (B) Scatter plots of Functional Status Score for the Intensive Care Unit with Medical Research Council-Sum Score and handgrip strength (dominant hand), Spearman correlation test, p value < 0.05. (C) and (D) Was ROC curves generated between significant association among the Functional Status Score for the Intensive Care Unit, the Medical Research Council-Sum Score and handgrip strength. (C) The cutoff values for the pre-ambulation score of the Functional Status Score for the Intensive Care Unit corresponded to a cutoff of 49 points for the Medical Research Council-Sum Score (sensitivity, 76.9%; specificity, 82.9%; negative predictive value, 90.6%; positive predictive value, 62.5%; precision, 81.3%). The cutoff for handgrip strength in the dominant limb was 16 kg/f (sensitivity, 83.3%; specificity, 63.2%; negative predictive value, 90.5%; positive predictive value, 47.6%; accuracy, 69%). (D) The cutoff values for the ambulation score of the Functional Status Score corresponded to a cutoff of 57 points for the Medical Research Council-Sum Score (sensitivity, 66.7%; specificity, 95.2%; negative predictive value, 95.2%; positive predictive value, 66.7%; precision, 91.7%). The cutoff for handgrip strength in the dominant limb was 18 kg/f (sensitivity, 83.3%; specificity, 61.1%; negative predictive value, 95.7%; positive predictive value, 26.3%; accuracy, 64.3%).

FSS-ICU - Functional Status Score for the Intensive Care Unit; HGS - handgrip strength; MRC-SS - Medical Research Council - Sum Score.

Associations among the FSS-ICU, MRC-SS and HGS and with other functional scales have been demonstrated in the literature.^(8-10)^ To date, no studies have evaluated the cutoff values for MS prediction in the FSS-ICU. However, one cohort study suggested a minimum cutoff of 41.5 out of 60 points on the MRC-SS as a predictor of the performance of the functional components of the Physical Function Intensive Care Test (PFIT) at ICU discharge.^(9)^ Our findings demonstrate that the FSS-ICU, MRC-SS and HGS are effective tools for measuring the progression of patient functionality in the ICU. In addition, the strength measures demonstrated a significant association with the functional independence of patients.

## Supplementary Material


