# Supplementary material for: Analysis of the Functional Status Score for the Intensive Care Unit and its correlation with measures of muscle strength in critically ill patients during hospitalization in the intensive care unit
Source: Crit Care Sci. 2025 Jan 30;37:e20250197. doi: 10.62675/2965-2774.20250197 (PMC11805452; doi:10.62675/2965-2774.20250197)
Supplement: Supplementary file 1 [file 2965-2774-ccsci-37-e20250197-suppl.pdf]

Critical Care Science
https://doi.org/10.62675/2965-2774.20250197

SUPPLEMENTARY MATERIAL

Analysis of the Functional Status Score for the Intensive Care Unit and its correlation with measures of muscle strength in critically ill patients during hospitalization in the intensive care unit

Gabriela de Sousa Martins[1], Katryne Holanda Silva[2], William Rafael Almeida Moraes[3], Eduardo Yoshio Nakano[4], Joanlise Marco de Leon Andrade[4], Laura Maria Tomazi Neves[3], Graziella França Bernardelli Cipriano[1,2]

METHODS

Study design/ethical aspects

This prospective cohort study followed the recommendations of the Strengthening Reporting of Observational Studies in Epidemiology Guidelines.(1) Evaluations were performed from March 2015 to July 2016 in an intensive care unit (ICU) for adults undergoing clinical and surgical treatment with 10 beds in a public hospital in the Federal District. This study was approved by the Committee for Ethics in Human Research (CAEE: 30442514.7.0000.5553). All patients and their legal guardians were informed about the study protocol and agreed to participate by signing an informed consent form.

Sample

All male and female patients aged ≥ 18 years who remained in the ICU for a period of ≥ 24 days were included. Patients with motor and/or neurological sequelae prior to hospitalization, those who died and those who did not complete all the evaluations were excluded.

The sample consisted of 48 patients (Figure 1S) who were recruited via a nonprobability convenience sampling technique. The minimum estimated sample size was 40 patients based on the assessments used, the Functional Status Score for the Intensive Care Unit (FSS-ICU) and Medical Research Council-Sum Score (MRC-SS); the measurements at awakening and at discharge from the ICU were compared. A clinically significant difference of half a standard deviation (effect size = 0.5), 95% significance level, 80% power and 20% dropout percentage was considered. A variation of 0.5 standard deviations corresponds to the mean effect size(2) and, according to Huang et al.,(3) represents a mean change of 3 points in the FSS-ICU.

Study procedures

Patients were monitored daily for neurological comprehension beginning upon awakening in the ICU, defined as the first day, via their responses to the following five commands: “open (close) your eyes”; “look at me”; “open your mouth and stick your tongue out”; “nod your head”; and “raise your eyebrows while counting to five”, which were described by De Jonghe et al.(4) Patients were considered fit for the assessments if they responded to three of these five commands in two consecutive assessments, with an interval of 6 hours. All patients underwent assessments of functional status and muscle strength. The evaluations were performed at awakening and at ICU discharge by a team of physical therapists previously trained in the use of the scales.

Functional status was assessed via the FSS-ICU scale.(5) The FSS-ICU is a scale developed for ICU patients and assesses five tasks: rolling over, transferring from a supine to a sitting position, transferring from a sitting to standing position, sitting on the edge of the bed, and walking.(6) Each task received a score ranging from 0 points (for patients unable to

This is an open access article under the CC BY license (https://creativecommons.org/licenses/by/4.0/).

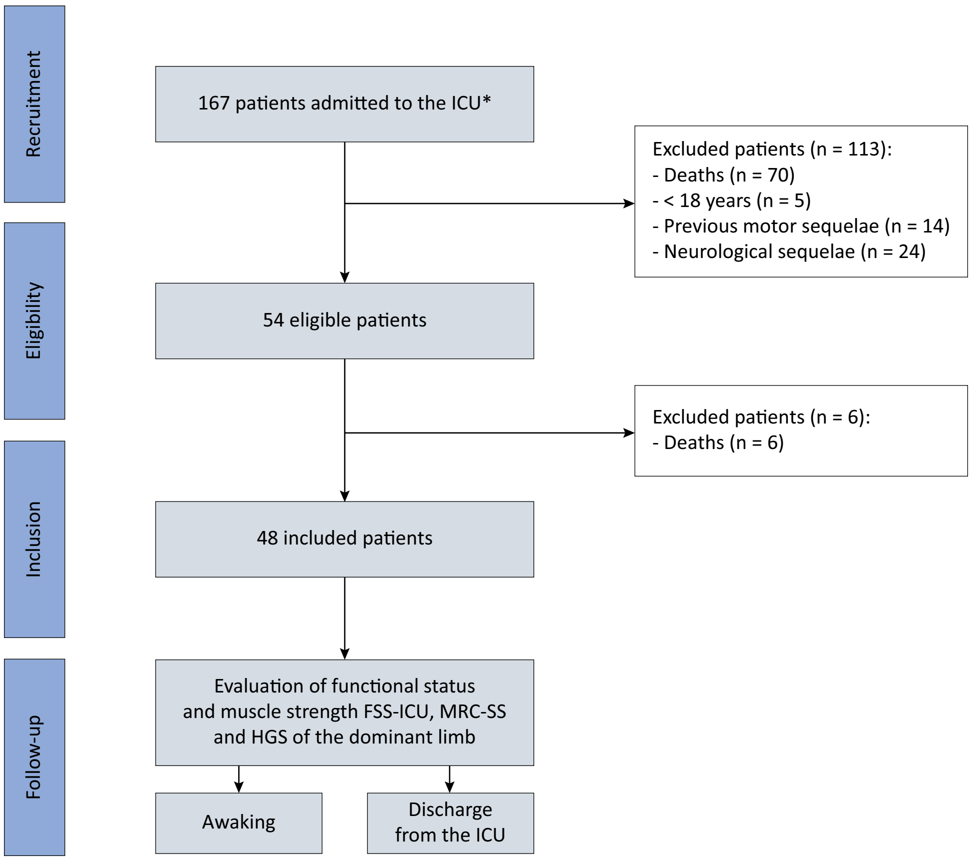

Figure 1S - Patient selection process.
* Period from March 2015 to July 2016 at the mixed intensive care unit of a public hospital.
ICU - intensive care unit; FSS-ICU - Functional Status Score for the Intensive Care Unit; MRC-SS - *Medical Research Council-Sum Score*; HGS - handgrip strength.

attempt or complete the task due to weakness) to 7 points (fully independent), with a cumulative score ranging from 0 to 35 points.[3,5,6]

Muscle strength was assessed via the MRC-SS scale and handgrip strength (HGS). The MRC-SS consists of a manual muscle strength test that assesses six bilateral muscle groups: shoulder abductors, elbow flexors, wrist extensors, hip flexors, knee extensors and dorsiflexors.[7] Each muscle group was scored from 0 points (paralysis) to 5 points (normal muscle strength), with the total score ranging from 0 to 60 points.[4,7] The evaluation was initially performed with the patient's limb positioned against gravity (grade 3). If the patient was able to perform the movement, isometric resistance was applied at the end of the range of motion to test muscle strength at grade 4 (active movement against gravity and light resistance) and grade 5 (movement against strong resistance - normal force), as described by Parry et al.[8] For patients who were unable to perform the initial movement initially against gravity, the position of the limb was modified in favor of gravity to test muscle strength grades from 0 (no visible contraction) to 2 (active movement with elimination of gravity).

Handgrip strength was assessed according to the recommendations of the American Society of Hand Therapists (ASHT).[9] Measurements were performed with a handheld digital dynamometer (Jamar Plus+ from Patterson Medical Ltd., Illinois, United States). The physical therapist supported the device manually during measurements.[7] Three measurements were performed bilaterally, and the highest value of the dominant limb was considered for analysis.

Additional clinical data were collected for sample characterization as follows: sex, age, severity index at admission measured by the Acute Physiology and Chronic Health Evaluation II (APACHE II), previous comorbidities, smoking, alcohol consumption, body mass index, causes of ICU admission, duration of mechanical ventilation (MV), days of MV, presence

of organ dysfunctions (sepsis, need for hemodialysis, hematological replacement and muscle weakness defined as MRC-SS ≤ 48 points), medications used (corticosteroids, sedatives, vasoactive drugs, antibiotics, diuretics and muscle blockers), duration of hospital stay before admission to the ICU, duration of stay in the ICU and days between admission and awakening.

Data analysis

Data normality was assessed via the Kolmogorov–Smirnov test with Lilliefors correction. Categorical data are presented as frequencies and proportions (%), and quantitative data are presented as the means and standard deviations (SDs) if normally distributed or medians and interquartile ranges (IQRs) if nonnormally distributed.

The measurements of the FSS-ICU, MRC-SS and HGS of the dominant limb at awakening and at discharge were compared via the paired Wilcoxon test. The floor and ceiling effects of the FSS-ICU and MRC-SS are expressed as percentages and were calculated by computing the number of participants who scored a minimum score (floor) of 0 for all the components or a maximum score (ceiling) of 35 points for the FSS-ICU and 60 points for the MRC-SS; the results are presented in table 1S.

The association between FSS-ICU score and muscle strength was determined via Spearman's correlation test and categorized as follows: $|rho| < 0.25$, none; $0.25 \leq |rho| < 0.5$, weak; $0.5 \leq |rho| < 0.75$, moderate; and $0.75 \leq |rho| \leq 1.0$, strong.[2]

To verify the value of muscle strength in independently predicting the ability to perform FSS-ICU tasks upon awakening, receiver operating characteristic (ROC) curves were constructed, and point and interval estimates (95% confidence intervals) of the area under the curve were estimated. In this study, the value at which the sum of sensitivity and specificity was maximized was determined as the "best" cutoff value for the MRC-SS score and handgrip strength. For this analysis, we categorized the FSS-ICU activities into two categories, with the pre-ambulation category consisting of three tasks, namely, rolling over, transferring from supine to the sitting position and sitting on the edge of the bed, and the ambulation category consisting of two tasks, namely, transferring from the sitting to the standing position and walking, following the same categorization proposed by Thrush et al.[6]

Independence was defined as the patient's ability to perform each of the FSS-ICU tasks with a score of 6 or 7 points. According to the study by Silva et al.,[5] scores of 6 and 7 points indicate modified independence and total independence, respectively. Thus, the final score for independence was ≥ 18 points for pre-ambulation tasks and ≥ 12 points for ambulation tasks.

A significance of 5% ($p < 0.05$) was used for all analyses, and the analyses were performed with the free software R, version 3.3.1, and the Statistical Package for the Social Sciences, version 23.

Table 1S - Floor and ceiling effects of the Functional Status Score for the Intensive Care Unit and Medical Research Council-Sum Score during hospitalization in the intensive care unit

Measures	Floor effect n/total n assessed (%)	Ceiling effect n/total n assessed (%)	Range of test score assessed
Awakening			
FSS-ICU	0/48 (0)	2/48 (4)	5 to 35 out of 35
MRC-SS	0/48 (0)	2/48 (4)	23 to 60 out of 60
Discharge from the ICU			
FSS-ICU	0/48 (0)	9/48 (19)	5 to 35 out of 35
MRC-SS	0/48 (0)	7/48 (15)	28 to 60 out of 60

FSS-ICU - Functional Status Score for the Intensive Care Unit; MRC-SS - Medical Research Council-Sum Score; ICU - intensive care unit.

REFERENCES

1. von Elm E, Altman DG, Egger M, Pocock SJ, Gøtzsche PC, Vandenbroucke JP; STROBE Initiative. The Strengthening the Reporting of Observational Studies in Epidemiology (STROBE) statement: guidelines for reporting observational studies. PLoS Med. 2007;4(10):e296.
2. Cohen J. Statistical Power analysis for the behavioral sciences. New York: Academic Press; 1969.
3. Huang M, Chan KS, Zanni JM, Parry SM, Neto JA, da Silva VZ, et al. Functional Status Score for the ICU: an international clinimetric analysis of validity, responsiveness, and minimal important difference. Crit Care Med. 2016;44(12):e1155-64.
4. De Jonghe B, Sharshar T, Lefaucheur JP, Authier FJ, Durand-Zaleski I, Boussarsar M, Cerf C, Renaud E, Mesrati F, Carlet J, Raphaël JC, Outin H, Bastuji-Garin S; Groupe de Réflexion et d'Etude des Neuromyopathies en Réanimation. Paresis acquired in the intensive care unit. a prospective multicenter study. JAMA. 2002;288(22):2859-67.
5. Silva VZ, Araújo JA Neto, Cipriano G Jr, Pinedo M, Needham DM, Zanni JM, et al. Brazilian version of the Functional Status Score for the ICU: translation and cross-cultural adaptation. Rev Bras Ter Intensiva. 2017;29(1):34-8.
6. Thrush A, Rozek M, Dekerlegand JL. The clinical utility of the Functional Status Score for the Intensive Care Unit (FSS-ICU) at a long-term acute care hospital: a prospective cohort study. Phys Ther. 2012;92(12):1536-45.
7. Hermans G, Clerckx B, Vanhullebusch T, Segers J, Vanpee G, Robbeets C, et al. Interobserver agreement of Medical Research Council Sum Score and handgrip strength in the intensive care unit. Muscle Nerve. 2012;45(1):18-25.
8. Parry SM, Berney S, Granger CL, Dunlop DL, Murphy L, El-Ansary D, et al. A new two-tier strength assessment approach to the diagnosis of weakness in intensive care: an observational study. Crit Care. 2015;19(1):52.
9. Fess EE. Grip strength. In: Casanova JS, editor. Clinical assessment recommendations. 2nd ed. Chicago: American Society of Hand Therapists; 1992. p. 41-5.
